# Validation of an Instrument for Measuring Adherence to Treatment With Immunomodulators in Patients With Multiple Myeloma

**DOI:** 10.3389/fphar.2021.651523

**Published:** 2021-05-05

**Authors:** Lívia Pena Silveira, Cristiane Aparecida Menezes de Pádua, Paula Lana de Miranda Drummond, Jéssica Soares Malta, Roberta Márcia Marques dos Santos, Naiane Lima Costa, Taísa Roberta Lopes Machado, Adriano Max Moreira Reis

**Affiliations:** ^1^ Hospital das Clínicas, Universidade Federal de Minas Gerais, Belo Horizonte, Brazil; ^2^ Faculdade de Farmácia, Universidade Federal de Minas Gerais, Belo Horizonte, Brazil; ^3^ Fundação Ezequiel Dias, Belo Horizonte, Brazil

**Keywords:** adherence., immunomodulators, multiple myeloma., adherence measures., validation

## Abstract

**Background:** Validate the Treatment Adherence Measure (TAM) instrument in outpatients with MM concerning construct validity, reliability and the ceiling and floor effects.

**Methods:** This cross-sectional study included patients diagnosed with MM previously treated with an immunomodulator for at least one month, aged 18 or over, and followed-up in the investigated outpatient clinics. Adherence to immunomodulators was measured by TAM. The TAM’s reliability was assessed using Cronbach’s alpha; The association between adherence and health-related quality of life was investigated to analyze the divergent and convergent construct, measured by the Quality of Life Questionnaire core (QLQ-C30) and the Quality of Life Questionnaire Multiple Myeloma module (QLQ-MY20). The presence of a ceiling or floor effect in the TAM was also analyzed.

**Results:** Eighty-four patients were included in the study, achieving 97.6% adherence. Cronbach’s alpha was 0.41, and the hypothesis of convergent construct validity was confirmed, with statistical significance, in contrast to the hypothesis of divergent construct validity. The presence of the ceiling effect in TAM suggested that this instrument does not allow changes to be detected in individuals concerning adherence to IMiDs.

**Conclusion:** TAM instrument did not show satisfactory validity and reliability to measure MM’s adherence. MM patients treated at oncohematological outpatient clinics in a metropolitan region of southeastern Brazil showed high adherence to IMiDs.

## Introduction

Multiple myeloma (MM) is an incurable neoplasm characterized by the unregulated proliferation of plasmocytes in the bone marrow, which is about 1% of all cancers in the world and the second most frequent hematological cancer (Picot et al., 2011; Anderson, 2016; Kazandjian, 2016; Cho et al., 2017; Holstein and Mccarthy, 2017; Kumar et al., 2017; Curado et al., 2018). As it is an incurable disease, the treatment of MM aims to increase the patient’s survival time and promote a better quality of life (Picot et al., 2011).

The goals of therapy in MM are to prolong overall survival, and to palliate symptoms and reduce or delay an organ damage, all while preserving quality of life. Therapeutic regimens generally containing either an alkylator, proteasome inhibitor or of immunomodulatory drug (IMiD), together with steroid, are currently standard-of-care for most patients (Hungria et al., 2019; Lu, 2020). A notable advance in MM treatment has been observed in the last two decades, with the introduction of oral immunomodulators (IMiDs): thalidomide, lenalidomide, and pomalidomide, which are widely used in clinical practice (Anderson, 2016; Holstein and Mccarthy, 2017; Cransac et al., 2019; Feiten et al., 2019; Hungria et al., 2019). In Brazil Thalidomide-based regimens are frequently prescribed (Hungria et al., 2019). Thalidomide is produced by a public foundation linked to the Brazilian Unified Health System (BUHS) and is dispensed free of charge only at public health system pharmacies accredited to care for MM patients. Lenalidomide has been registered in Brazil; however, it is not available to treatment free of charge in BUHS services (Silveira et al., 2021).

Adherence to treatment is crucial for the treatment’s success with oral antineoplastic agents, such as IMiDs (Mislang et al., 2017). However, there is currently no gold standard method for measuring treatment adherence. Direct methods, such as the dosage of the active ingredient or its metabolite in blood or urine, are more expensive and barely used in clinical practice or scientific studies. Indirect methods, in turn, are simpler to apply, low cost and non-invasive, among which the self-report scales stand out (Osterberg and Blaschke, 2005; Gimenes et al., 2009).

The self-report adherence measure using scales can achieve cross-cultural validation and psychometric analysis, contributing to its improvement (Stirratt et al., 2015). Instruments for measuring adherence to oral antineoplastics specific to MM are not available in Brazil. The Morisky Medication Adherence Scale (MMAS) was adapted and validated for Brazilian Portuguese, and a study of adherence to treatment in patients with MM was performed. However, no validation study was identified for these patients (Oliveira-Filho et al., 2012; Gupta et al., 2018).

Also, MMAS started to demand copyright, hindering its use in research in developing countries. The Treatment Adherence Measure (TAM) instrument was developed in Portugal, validated in Brazil in patients with noncommunicable diseases, and is exempt from copyright (Carvalho et al., 2010; Boas et al., 2014; Hungria et al., 2019). No published studies on adherence to oral antineoplastics using TAM have been identified to date.

Given the relevance of adherence to oral antineoplastic agents for treating hematological neoplasms, self-report measures that are valid, reliable, easy to apply, and efficient are essential (Vrijens et al., 2012; Daouphars et al., 2013; Stirratt et al., 2015; Cransac et al., 2019). Thus, this study aimed to validate TAM in outpatients with MM concerning the construct’s validity, reliability, and the ceiling and floor effects.

## Methods

### Study Design and Location

This is a methodological, monocentric, cross-sectional study carried out from April 2019 to March 2020 in oncohematological outpatient clinics in the public and private network of the third-largest metropolitan region in southeastern Brazil. The study included patients diagnosed with MM previously treated with immunomodulators for at least one month, aged 18 years or older, and followed-up in the investigated outpatient clinics. Patients who met these inclusion criteria and voluntarily accepted the research signed the informed consent form. The Research Ethics Committee approved the study under CAAE 05400818.3.0000.5149 and 05400818.3.3004.5119.

### Patients

The sample encompassed all patients identified with MM diagnosis from outpatient clinics and attended an appointment during the study period. All patients were invited to participate. The outpatient clinics were located in Belo Horizonte, namely, two outpatient clinics in the public network and one clinic in the private network. A single face-to-face interview was performed with the participants using adherence and Health-related Quality of Life (HRQoL) instruments, the questions were read by the researchers. The interview had also sociodemographic and clinical questions. Patients were interviewed at different phases of treatment. Therefore, there was no pattern of time to start treatment with IMiDs and the day of the interview. Patient medical records were assessed to complement clinical data. The interview data was registered by researcher using *Questionnaire Development System* (QDS, version 2.6.1.1).

### Treatment Adherence Measure Instrument

The TAM was developed and validated in Portugal by (Delgado and Lima, 2001). The instrument has good internal consistency, sensitivity, and specificity. It consists of seven questioning items for adherence measures that encompass the patient’s behavioral and economic issues, besides the characteristics of the prescription itself (Delgado and Lima, 2001). TAM items, has semantic, idiomatic, cultural coherence with Brazilian Portuguese (Borba et al., 2018). A written authorization to use TAM in this research was obtained.

Answers to the seven items are obtained using a six-point ordinal scale, ranging from one to six, with 1 = always, 2 = almost always, 3 = frequently, 4 = sometimes, 5 = rarely, and 6 = never. The level of adherence is obtained by adding the values of each response (ranging from 1 to 6) and dividing by the total number of items, and the value found is converted into a dichotomous variable (adherence and non-adherence). Individuals who obtain the arithmetic mean values from one to four, are considered as non-adherent. For those achieving a final score between five and six are considered adherent (Delgado and Lima, 2001). In this study, the TAM was adapted to the research objectives, replacing the expression “medicines for the disease” with the “immunomodulator that the patient used”. The mean time needed to complete TAM was three minutes.

### Variables and Data Collection

#### Adherence

Adherence to immunomodulators was measured with TAM (Delgado and Lima, 2001). Adherence to drug treatment is the process in which the patient uses the drug as prescribed. The adherence phase measured in this study was the implementation, which refers to the extent to which the patient’s dose corresponds to the prescribed therapeutic regimen, covering from the first dose to the last dose of the medication (Vrijens et al., 2012; De Geest et al., 2018).

#### Health-Related Quality of Life

HRQoL was measured using the multidimensional instruments Quality of life questionnaire core (QLQ-C30) and Quality of life questionnaire multiple myeloma module (QLQ-MY20) (EORTC, 2001; EORTC, 2007). The QLQ-C30 was developed to assess HRQoL in cancer patients and covers overall health status and quality of life, functional scale, and symptom scale. The QLQ-MY20 is a specific instrument for assessing HRQoL in patients with MM and covers the scales of side effects, disease symptoms, body image, and future perspective. These instruments were developed by the European Organization Research Treatment of *Cancer* (EORTC) (EORTC, 2001; EORTC, 2007).

HRQoL was assessed using QLQ-C30 in association QLQ-MY20, as recommended. The use of the instruments was authorized by the quality of life group of the European Organization for Research and Treatment of *Cancer* (EORTC), responsible for the elaboration and availability of the instruments. It was exempt from copyright because it is research in an academic scope (EORTC, 2019).

The QLQ-C30 instrument contains 30 assessment items divided into three main domains, global health status and quality of life, symptom scales, and functional scales (Aaronson et al., 1993). The QLQ-C30 was validated for Brazil and applied to MM patients in the country (Etto et al., 2011; Paiva et al., 2014).

The QLQ-MY20 module is used in a complementary way QLQ-C30, and contains 20 specific questions for MM, divided into two main domains. The first is the symptom scales, which have disease symptoms scales and side effects to treatment. The second domain is functional scales, divided into body image and future perspective. The specific module QLQ-MY20 was recently validated in Brazilian patients with adequate psychometric properties showing validity and reliability of the instrument (Malta et al., 2020).

The scores for each domain or scale of QLQ-MY20 or QLQ-C30 were calculated from the responses obtained through the instruments, according to the manual recommended by the EORTC group (EORTC, 2001; EORTC, 2007). The scores were achieved through a linear transformation, reaching scores ranging from 0 to 100 at the end. Scores were interpreted according to the scales–high scores on the symptom domain scales represent high symptomatology levels or side effects to treatment, while high scores on the functional scales represent high functionality (EORTC, 2001; EORTC, 2007).

#### Sociodemographic, Clinical, Care, and Pharmacotherapeutic Variables

Information on sociodemographic variables (age, gender, skin color, income, schooling, and occupation) was obtained through face-to-face interviews with patients on the day of the scheduled appointment at the outpatient clinic. Patients were also asked if they need some help to take their medication. Other clinical, care, and pharmacotherapeutic variables were collected through interviews and supplemented by collecting medical records data. Polypharmacy was defined as using five or more medications by the patient, and multimorbidity, as the simultaneous presence of two or more chronic diseases (Ramos et al., 2016; Nunes et al., 2018).

### Data Analysis

The descriptive analysis was performed by determining the frequencies for categorical variables and measures of central tendency and dispersion measures for continuous variables. The variables were evaluated concerning the normal distribution using the Kolmogorov-Smirnov test.

The instrument’s reliability, assessed by the TAM items’ internal consistency, was verified using Cronbach’s alpha. The standard value for high internal consistency was α> 0.70, and α around 0.50 was acceptable for scales with few items. The item-total correlation, that is the correlation between the item and the instrument’s total score, was also calculated to verify whether the items discriminate patients concerning adherence. Values below 0.3 indicate that the item does not contribute to this discrimination (de Vet et al., 2011). The mean and standard deviation of responses for the instrument and each item were also determined.

Concerning the convergent construct validity analysis, the hypothesis investigated was a moderate association between adherence measured by TAM and HRQoL measured by the global health status and quality of life domains of the QLQ-C30. Regarding the analysis of divergent construct validity, the hypothesis was no association between adherence measured by TAM and HRQoL measured by the symptom scale domains of QLQ-C30 and side effects and symptoms of disease measured by QLQ-MY20. The correlation was assessed using the Spearman’s correlation test. The values are classified as having little clinical applicability (less than 0.3), moderate magnitude (between 0.3 and 0.5), and substantial magnitude (above 0.5) (Carvalho et al., 2010). The construct validity analysis was based on the study developed by Gupta et al. (2018), which showed an association between higher adherence and better quality of life (Gupta et al., 2018).

Concerning the analysis of the ceiling and floor effect, the floor effect was considered when more than 15% of the respondents opted for the response of value 1 and ceiling when more than 15% of the respondents opted for the response of value 6 (Terwee et al., 2007). Statistical analysis was performed using the Statistical Package for Social Sciences® (SPSS®) software, version 25.0.

## Results

A total of 84 patients were included in the study. Of these, 50% were male, 59.5% self-declared black, and the median age was 62.7 years (IQR = 14.4). More than half of the patients (58.3%) underwent treatment in the public health system, and half had two or more comorbidities. The other characteristics of these patients are listed in Table 1.

**TABLE 1:** Sociodemographic, care, clinical and pharmacotherapeutic characteristics of patients with Multiple Myeloma using immunomodulators (*n* = 84).

Characteristics	Values	
**Sociodemographic**		
**Age in years [median (interquartile range–IQR)]**	62.7	14.4
**Older adult [n, (%)]**	55	65.5
**Gender**		
Male [n, (%)]	42	50.0
**Skin color**		
Black [n, (%)]	50	59.5
**Income**		
Three minimum wages and over [n, (%)]	49	58.3
**Schooling**		
Up to 11 years of study [n, (%)]	52	61.9
**Occupation**		
Retired/not working [n, (%)]	65	77.4
**Care**		
**Place of treatment**		
Public [n, (%)]	49	58.3
**Hospitalization [n, (%)]**	36	42.9
**Diagnosis time in months [median (interquartile range–IQR–months)]**	15	31.8
**Clinical**		
**International staging system–ISS**		
I	24	28.6
II	20	23.8
III	27	32.1
Absent	*13*	*15.5*
**Myeloma multiple stages**		
**Induction**	*40*	*47,6*
**Consolidation**	*4*	*4,8*
**Maintenance**	*15*	*17,9*
**Remission**	*25*	*29,8*
**Hematopoietic stem cell transplantation [n, (%)]**		
Yes	28	33.3
No	35	41.7
Absent	21	25.0
**Comorbidities**		
Arterial hypertension	52	61.9
Diabetes	11	13.1
Cancer	13	15.5
Chronic kidney disease	11	13.1
Cardiovascular	11	13.1
**Multimorbidity [n, (%)]**	42	50.0
**Pharmacotherapeutic**		
**Polypharmacy [n, (%)]**	67	79.8
**Current treatment regimens**		
Thalidomide	9	10.7
Thalidomide + corticosteroids/alkylators^a^	44	52.5
Thalidomide + proteasome inhibitor + corticoid/alkylator^b^	19	22.6
Lenalidomide	6	7.1
Lenalidomide + corticoid^c^	3	3.6
Lenalidomide + proteasome inhibitor + corticoid^d^	2	2.4
Pomalidomide + corticoids/alkylators^e^	1	1.2
**Adverse reactions reported in the medical record [n, (%)]**	82	97.6
Constipation	39	46.4
Drowsiness	6	7.1
Thrombosis	4	4.8
Peripheral neuropathy	54	64.3

^a^ CTD (cyclophosphamide + thalidomide + dexamethasone), DT (dexamethasone + thalidomide), MPT (melphalan + prednisone + thalidomide)

^b^ VTD (bortezomib + thalidomide + dexamethasone), VMPT (bortezomib + melphalan + prednisone + thalidomide), KTD (carfilzomib + thalidomide + dexamethasone)

^c^ RD (lenalidomide + dexamethasone)

^d^ KRD (carfilzomib + lenalidomide + dexamethasone)

^e^ CPD (cyclophosphamide + pomalidomide + dexamethasone).

Approximately 85% of patients used thalidomide, followed by 13.1% who used lenalidomide and 1.2%, pomalidomide. Moreover, 97.6% had an adverse event recorded in the medical records (Table 1). The most frequent treatment regimens among patients contained thalidomide, an alkylating agent, or a corticosteroid, such as CTD (cyclophosphamide, thalidomide, dexamethasone), DT (dexamethasone, thalidomide), and MPT (melphalan, prednisone, thalidomide) (Table 1).

Most patients answered “never” or “rarely” to TAM questions, which resulted in 97.6% adherence and most patients did not have a caregiver to administer their medications (78,5%). The descriptive analysis of TAM items is described in Table 2 and shows an average value of 5.7 for the scale score and mean values of 5.3–5.9 for the items.

**TABLE 2:** Descriptive analysis of the treatment adherence measure (TAM) instrument in patients using an immunomodulator (*n* = 84).

Treatment adherence measure (TAM) items	Mean (SD)	Median (IQR)	Minimum - maximum	Adjusted item-total correlation	Cronbach’s alpha if the item is excluded
1. Have you ever forgotten to take the medications for your illness?	5.6 (0.8)	6.0 (0.0)	1.0–6.0	0.4	0.2
2. Have you ever been careless with the hours of taking medications for your illness?	5.3 (1.3)	6.0 (1.0)	1.0–6.0	0.3	0.3
3. Have you ever stopped taking medications for your illness because you felt better?	5.9 (0.4)	6.0 (0.0)	2.0–6.0	0.1	0.4
4. Have you ever stopped taking the medicines for your illness, on your initiative, after feeling worse?	5.9 (0.3)	6.0 (1.0)	4.0–6.0	0.2	0.4
5. Have you ever taken one or more pills for your illness, on your initiative, after feeling worse?	5.9 (0.1)	6.0 (0.0)	5.0–6.0	-0.0	0.4
6. Have you ever interrupted therapy for your illness because you missed the medication?	5.7 (0.7)	6.0 (0.0)	2.0–6.0	0.2	0.4
7. Have you ever stopped taking medications for your illness for any reason other than the doctor’s recommendation?	5.9 (0.3)	6.0 (0.0)	4.0–6.0	0.2	0.4
Total scale	5.7 (0.3)	5.8 (0.3)	4.6–6.0	−	−

Table 3 lists the distribution of the percentage of responses to each TAM item. A maximum effect is observed in the studied sample since the values for the answer “never” are above 15% (Carvalho et al., 2010).

**TABLE 3:** Distribution of the percentage of responses to the questions of the Treatment Adherence Measure (TAM) instrument elaborated by patients using immunomodulator (*n* = 84).

Treatment adherence measure (TAM) items	Always	Almost always	Frequently	Sometimes	Rarely (%)	Never (%)
1. Have you ever forgotten to take the medications for your illness?	1.2%	−	1.2%	4.8%	16.7	76.2
2. Have you ever been careless with the hours of taking medications for your illness?	3.6%	3.6%	−	10.7%	19.0	63.1
3. Have you ever stopped taking medications for your illness because you felt better?	−	1.2%	−	−	1.2	97.6
4. Have you ever stopped taking the medicines for your illness, on your initiative, after feeling worse?	−	−	−	1.2%	2.4	96.4
5. Have you ever taken one or more pills for your illness, on your initiative, after feeling worse?	−	−	−	−	1.2	98.8
6. Have you ever interrupted therapy for your illness because you missed the medication?	−	1.2%	−	7.1%	13.1	78.6
7. Have you ever stopped taking medications for your illness for any reason other than the doctor’s recommendation?	−	−	−	1.2%	6.0	92.9

Regarding the reliability of the instrument, the value obtained from Cronbach’s alpha was 0.41. This value has a downward trend when each item is removed, ranging from 0.2 to 0.4. Each item’s correlations with the total scale were considered adequate only for items 1 and 2 (Table 2) (de Vet et al., 2011).

No correlation was identified between adherence measured by TAM and the side effects domain by QLQ-MY20 (rho = -0.048; *p* = 0.666). Low magnitude correlation was detected between adherence measured by the TAM and the global health status domain of the QLQ-C30 (rho = 0.254; *p* = 0.020).

## Discussion

This study is innovative in investigating the validity of a self-report instrument for measuring adherence in outpatients with MM in Brazil. The investigation of the validity of a self-report instrument in measuring medication adherence for use in patients with MM has international relevance. Valid and reliable instruments will contribute to more accurate measures and increased knowledge about the magnitude of non-adherence to treatment with IMiDs in clinical practice. Moreover, they also provide robust evidence to promote interventions aimed at streamlining adherence and the effectiveness of MM treatment (Cransac et al., 2019; Ross et al., 2020).

In Brazil, the thalidomide immunomodulator is available for free to patients in the public and private system and is currently produced by a single public laboratory (Silveira et al., 2021). Dispensing to patients is only made in pharmacies in the public health system accredited to assist patients using MM (BRASIL, 2011; Silveira et al., 2021). The easy access to the medication explains the greater use of this IMiD by the study patients. On the other hand, the newer IMiDs are only available to patients who undergo treatment in private services and have the financial means to acquire them.

As a result, most treatment regimens used by patients cover thalidomide, with more than half using thalidomide associated with a corticosteroid or alkylator. This is because all these drugs are provided by the Brazilian public health system, unlike bortezomib, which is also part of MM’s treatment lines but is not available in the country’s public health system. In the study by Hungria et al., 2019, thalidomide treatment was also the most frequent in Brazil and other Latin American countries (Hungria et al., 2019).

Adherence to IMiDs was high in the patients of the investigated outpatient clinics. However, TAM’s evaluated properties in patients with MM did not confirm the validity and reliability of the instrument identified in patients with diabetes, hypertension, and mental health (Boas et al., 2014; Borba et al., 2018). Is needed to clarify that TAM applies to many clinical and therapeutic contexts (Delgado and Lima, 2001), and although developed in Portugal the sociocultural context was similar the present study. Consequently, the performance of validity and reliability in MM need be better investigated adding in the research particular issues related to cancer treatment to support the use in the evaluation of adherence in MM patients. The instrument’s reliability, considering Cronbach’s alpha of 0.41, was not satisfactory, since it is outside the appropriate range of 0.70 and 0.90. Cronbach’s alpha values in the original study were 0.74 and varied from 0.6 to 0.84 in studies with chronic diseases in Brazil (Delgado and Lima, 2001; Carvalho et al., 2010; Boas et al., 2014). It is worth mentioning that Cronbach’s alpha depends on the number of items and that scales with few items, such as TAM, can have Cronbach’s alpha values around 0.5 that are acceptable (Nunnally and Bernstein, 1994; Bowling, 2005). Other studies that used questionnaires with a reduced number of items and in the dichotomous form reported values between 0.5 and 0.6 (Carvalho et al., 2010; Boas et al., 2014).

Cronbach’s alpha assesses the instrument’s internal consistency, which can also be verified by the value assumed by Cronbach’s alpha after item exclusion. The increase in the significant value of one item is indicative that the item is not sufficiently consistent with the others, which did not occur in this study with patients with MM (Gasparin et al., 2010). Only items 1 and 2 of the instrument can distinguish between patients with low and high adherence, as they had an item-total correlation ≥0.3. The others did not contribute much to this distinction and may be items that can be excluded (de Vet et al., 2011).

Although previous study has shown an association between adherence measured by Morisky-Green and the patient’s Health Status Measure and quality of life using the Functional Assessment of *Cancer* Therapy–Multiple Myeloma, the convergent construct validity has not been confirmed because the association between TAM adherence and the domain of the overall health status and quality of life of the QLQ-C30 had a low magnitude (Gupta et al., 2018). This can be explained by the fact that TAM measures domains, such as barriers to treatment and medication use behavior, impact the effectiveness and, consequently, the quality of life. However, the domains are not fully measured in the overall health status subscale (QLQ-30).

The hypothesis of divergent validity was confirmed, but it did not show statistical significance. The result showed the absence of an expected association, that is, patients with higher adherence showed less intensity of symptoms of the disease and more manifestations of side effects of the drug. In addition, TAM does note measure symptoms of the disease, it only addresses side effects as a barrier to adherence. It is worth noting that Gupta et al. (2018), emphasize that adherence contributes to reducing the burden of the disease in patients with MM. Thus, the hypothesis that the higher the adherence to treatment with oral antineoplastic, the lower the report of symptoms, is feasible because one of the objectives of treatment with IMiDs is the reduction of the symptoms of the disease (Gupta et al., 2018).

The interpretability of an instrument’s score is not a measurement property, but an essential requirement for the proper use of a measurement instrument and corresponds to the extent to which qualitative meaning can be attributed to the scores or their changes. The distribution of scores in the studied population can reveal a grouping of scores, which usually occurs at the extremes of the scale and indicates the lack of patients’ discriminatory capacity in this range of the scale, triggering the ceiling or floor effects. In most cases, these effects occur when the measurement instrument is applied to another target population other than the one for which it was initially developed, which occurred in this study and that of (Carvalho et al., 2010; de Vet et al., 2011). The presence of ceiling or floor effects minimizes the instrument’s sensitivity to detect changes. Therefore, in the case of MM patients, the detected ceiling effect suggests that TAM does not allow detecting changes in individuals concerning adherence to IMiDs (de Vet et al., 2011; Rodrigues et al., 2013).

The frequency of adherence to treatment with IMiDs using TAM was high. The small number of non-adherents compromised the analysis of the comparison between the groups. However, despite the sample size, the study’s individuals reflect the epidemiological profile of MM described in studies conducted in Brazil and Latin America concerning sociodemographic, clinical, and care characteristics (Hungria et al., 2019).

The high adherence to treatment with IMiDs is in line with studies in France and Germany (Cransac et al., 2019; Feiten et al., 2019). The French study evaluated a validated instrument for patients with chronic myeloid leukemia in measuring the adherence of patients with MM and found no reproducible results in comparison with the measurement by medication possession ratio (MPR), which highlights the demand for investigations for the development and validation of new instruments for measuring MM adherence.

It is important to highlight as a strength of our study the heterogeneous sample, composed of patients from the public and private health system in southeastern Brazil and in various MM stages (induction, consolidation, maintenance and remission). Moreover, the study was conducted in compliance with ESPACOMP’s Medication Adherence Reporting Guideline (EMERGE) (De Geest et al., 2018).

However, a limitation of this research is its conduction in a single metropolitan region of the country, which hinders generalization of the results. Another limitation could be the overestimation of adherence due to social desirability bias, which reflects people’s propensity to provide more socially acceptable answers, positively distorting the image of adherence to the medication. The reliability analysis was limited to internal consistency due to operational issues related to the difficulty of reconciling the application of the retest with the scheduled return of the patient to the clinic. Finally, our study was limited to evaluate psychometric parameters of validity and reliability, other analyses (e.g., confirmatory factor analysis, theory response item) are stimulated with the aim to better understand the performance of TAM in MM patient. So, future studies with higher number of patients are essential to achieve a best evidence to use TAM to support measure adherence in MM patients.

## Conclusion

The TAM instrument did not present satisfactory validity and reliability for measuring MM adherence to be used in clinical practice. MM patients treated at oncohematological outpatient clinics in a metropolitan region of southeastern Brazil showed high adherence to IMiDs.

## Data Availability

The raw data supporting the conclusions of this article will be made available by the authors, without undue reservation.
